# *In vivo* scanning laser fundus and high-resolution OCT imaging of retinal ganglion cell injury in a non-human primate model with an activatable fluorescent-labeled TAT peptide probe

**DOI:** 10.1371/journal.pone.0313579

**Published:** 2024-12-06

**Authors:** Xudong Qiu, Seth T. Gammon, Carol Rasmussen, Federica Pisaneschi, Charlene B. Y. Kim, James Ver Hoeve, Steven W. Millward, Edward M. Barnett, T. Michael Nork, Paul L. Kaufman, David Piwnica-Worms

**Affiliations:** 1 Department of Cancer Systems Imaging, The University of Texas MD Anderson Cancer Center, Houston, Texas, United States of America; 2 Department of Ophthalmology and Visual Sciences, University of Wisconsin School of Medicine and Public Health, Madison, Wisconsin, United States of America; 3 Department of Ophthalmology & Visual Sciences, Medical College of Wisconsin, Milwaukee, Wisconsin, United States of America; Transilvania University of Brasov: Universitatea Transilvania din Brasov, ROMANIA

## Abstract

The optical imaging agent TcapQ488 has enabled imaging of retinal ganglion cell (RGC) injury in vivo in rodents and has potential as an effective diagnostic probe for early detection and intervention monitoring in glaucoma patients. In the present study, we investigated TcapQ488 in non-human primates (NHPs) to identify labeling efficacy and early signals of injured RGC, to determine species-dependent changes in RGC probe uptake and clearance, and to determine dose-limiting toxicities. Doses of 3, 6, and 12 nmol of TcapQ488 were delivered intravitreally to normal healthy NHP eyes and eyes that had undergone hemiretinal endodiathermy axotomy (HEA) in the inferior retina. Post-injection fundus fluorescence imaging using a Spectralis imaging platform (Heidelberg Engineering) documented TcapQ488 activation in RGC cell bodies. Optical coherence tomography (OCT), slit-lamp examinations, intraocular pressure measurements, and visual electrophysiology testing were performed to monitor probe tolerability. For comparison, a negative control, non-cleavable, non-quenched probe (dTcap488, 6 nmol), was delivered intravitreally to a normal healthy eye. In normal healthy eyes, intravitreal injection of 3 nmol of TcapQ488 was well-tolerated, while 12 nmol of TcapQ488 to the healthy eye caused extensive probe activation in the ganglion cell layer (GCL) and eventual retinal nerve fiber layer thinning. In HEA eyes, the HEA procedure followed by intravitreal TcapQ488 (3 nmol) injection resulted in probe activation within cell bodies in the GCL, confined to the HEA-treated inferior retina, indicating cell injury and slow axonal transport in the GCL. However, in contrast to rodents, a vitreal haze that lasted 2–12 weeks obscured rapid high-resolution imaging of the fundus. By contrast, intravitreal TcapQ488 injection prior to the HEA procedure led to minimal probe labeling in the GCL. The results of the dTcap488 control experiments indicated that fast axonal transport carried the probe out of the retina after cell body uptake. No evidence of pan-retinal toxicity or loss of retino-cortical function was detected in any of the three NHPs tested. Overall, these data provide evidence of TcapQ488 activation, without toxicity, in NHP HEA eyes that had been intravitreally injected with 3 nmol of the probe. Compared to rodents, unexpectedly rapid axonal transport in the NHPs reduced the capacity to visualize RGC cell bodies and axons through the backdrop of an intravitreal haze. Nonetheless, although intravitreal clearance rates did not scale to NHPs, HEA-induced reductions in axonal transport enhanced probe visualization in the cell body.

## Introduction

Optical coherence tomography (OCT) is a non-invasive, standard-of-care imaging procedure that monitors anatomical structures throughout distinctive retina layers [1–3]. In primates, retinal ganglion cell (RGC) health can be accessed by measurement of the thickness of the peripapillary retinal nerve fiber layer (RNFL) and ganglion cell/inner plexiform layer (GCIPL) [4]. However, such morphology-based analysis by OCT of RNFL and GCIPL thinning are merely surrogate measurements for estimation of RGC loss, and lack sensitivity for early changes of function. Furthermore, while adaptive optics-OCT may provide information on RGC density and cell diameter changes within macular regions, the exam is performed in a relatively limited field-of-view and under significant acquisition time [5, 6]. Thus, in vivo molecular imaging of biochemical events preceding RGC loss would be ideal for early detection and interventional monitoring of RGC injury [7, 8].

TcapQ488, an optical molecular imaging permeation peptide agent, has enabled imaging *in vivo* of RGC injury in rodents and has potential as an effective technique in glaucoma patients. TcapQ488 has been shown to effectively detect effector caspase enzyme activation *in vitro*, *in cellulo*, and *in vivo* via fluorescence imaging [8–18]. Specifically, TcapQ488 consists of a modified TAT sequence (all D-amino acids RKKRRORRR), and an effector caspase-cleavable sequence (L-amino acids DEVD), flanked by an optically activatable quencher (QSY 7) and fluorophore (Alexa Fluor 488) pair that matches fluorescent signal detection availability on many clinical ophthalmology instruments, such as the Heidelberg Engineering Spectralis ophthalmic imaging platform. Moreover, dTcap488, composed of all D-amino acids, is a non-cleavable and non-quenched constitutive fluorescent peptide and can serve as a control probe to monitor cellular uptake. Further investigation of TcapQ488 *in vivo* in a non-human primate (NHP) model is a critical translational step in moving this diagnostic molecular imaging agent forward toward human clinical usage.

Hemiretinal endodiathermy axotomy (HEA) is a robust, reproducible, and reliable model of partial RGC axotomy that was developed in NHPs [19, 20]. The HEA procedure induces marked thinning of the RNFL and GCIPL in the inferior retina, but there is no secondary degeneration of RGCs in the superior retina. The outer nuclear layer, photoreceptor layer, and regional choroidal blood flow are not affected by HEA in either the inferior or superior retina.

The RNFL and GCIPL thinning in the HEA model is caused by direct RGC axon injury, leading to caspase activation at the RGC body. Therefore, we investigated whether TcapQ488 could be endocytosed by NHP RGCs and able to detect effector caspase activity in RGCs in an NHP model of HEA. We intravitreally injected TcapQ488 into NHPs after the HEA procedure. We expected that TcapQ488 would be sequestered by RGCs due to the cell permeation and nuclear localization features of the modified TAT sequence, where it would be cleaved by activated effector caspases at the DEVD sequence and release Alexa Fluor 488 to emit a fluorescent signal in the RGC cytoplasm.

## Materials and methods

### Animals and housing conditions, feeding regimens, environmental enrichment, anesthesia, disposition

Three adult cynomolgus macaques (*Macaca fascicularis*), 2 males and 1 female aged 10 to 15 years and weighing 5 to 9 kg, were used in this study (Table 1). The animals were designated Cy0407, Cy0410, and Cy0302.

**Table 1:** Hemiretinal endodiathermy axotomy and intravitreal probe injection procedures by date for all 3 non-human primates involved in the study.

Procedure	Cy0407, Male 13 years old		Cy0410, Male 15 years old		Cy0302, Female 10 years old	
	OD	OS	OD	OS	OD	OS
HEA			11/3/15			
3 nmol TcapQ488	6/16/15		11/10/15			
6 nmol TcapQ488	8/31/15		12/7/15		1/22/16	
6 nmol dTcap488				2/22/16		
12 nmol TcapQ488		2/22/16				
HEA					1/25/16	

Before the experiments began, all 3 animals were determined to be ocularly normal and systemically healthy. All of the experimental methods and techniques adhered to the ARVO Statement for the Use of Animals in Ophthalmic and Vision Research and were approved by the University of Wisconsin-Madison Animal Care and Use Committee. The macaques used in this study were cared for by the staff at the Wisconsin National Primate Research Center in accordance with recommendations of the Weatherall report and the principles described in the National Research Council’s Guide for the Care and Use of Laboratory Animals. Animals were housed in enclosures with the required floor space, with a 12-hour-light–dark cycle and room temperature of 21°C, and fed using a nutritional plan based on recommendations published by the National Research Council. Animals were fed an extruded dry diet (commercial NHP chow 2050 Teklad Global 20% Protein Primate Diet, Harlan Laboratories) twice daily, with adequate carbohydrates, energy, fat, fiber, mineral, protein, and vitamin content. Diets were supplemented with fruits, vegetables, and other edible objects (e.g., nuts, cereals, seed mixtures, yogurt, peanut butter, popcorn, marshmallows, etc.) to provide variety and to inspire species-specific behaviors such as foraging. To promote psychological well-being, animals were provided with food enrichment, structural enrichment, and/or manipulanda. Environmental enrichment objects were selected to minimize the chances of pathogen transmission from one animal to another and from animals to care staff. While on the study, all animals were evaluated by trained animal care staff at least twice daily for signs of pain, distress, and illness by observing appetite, stool quality, activity level, and physical condition. Animals exhibiting abnormal presentation for any of these clinical parameters were provided appropriate care by attending veterinarians. Before all minor/brief experimental procedures, macaques were sedated using ketamine anesthesia (details below) and vital signs (heart rate, blood oxygen saturation, respiratory rate, and temperature) were monitored until the animals fully recovered. Post procedure analgesia consisted of meloxicam 0.1 mg/kg subcutaneous or oral for up to 7 days and buprenorphine (up to 0.3mg/kg IM) every 6–12 hours for up to 3 days. At the end of the study, all 3 animals were deeply anesthetized with sodium pentobarbital (≥ 25 mg/kg intravenous) and transcardially perfused with heparinized saline, followed by 4% paraformaldehyde. A full necropsy of all animals was performed by a veterinary pathologist board-certified by the American College of Veterinary Pathologists. At baseline screening, animals underwent thorough eye examinations with both slit lamp examination and indirect ophthalmoscopy, intraocular pressure measurements, full-field electroretinography (ffERG) and visual evoked cortical potential (VEP) testing, fundus fluorescence angiography mode (FAM) imaging and optical coherence tomography (OCT) imaging.

All anesthetic regimens started with 10–15 mg/kg intramuscular (IM) ketamine hydrochloride (HCl). Full-field electroretinography /VEP and imaging (fundus photography, FAM, and OCT) included supplementation with ketamine (1–10 mg/kg, IM), as needed, and dexmedetomidine HCl (0.3 mg/kg, IM) to ensure immobility, non-responsiveness to tactile stimulation, cessation of the corneal blink reflex, and physiological stability. After ffERG/VEP and imaging, dexmedetomidine HCl sedation was reversed with atipamezole (0.3 mg/kg IM). Animal heart rate, percent oxygen saturation (SpO_2_), respiration rate, and rectal temperature were monitored and recorded throughout the experimental procedure. Core body temperature was maintained between 37°-39°C with a water-circulating heating pad. Animals were intubated and anesthetized via inhalation with an oxygen/isoflurane mix (3%-5% isoflurane for induction and 1%-3% isoflurane for maintenance) to sustain a modestly deep level of anesthesia during the HEA procedure. Pupils were dilated with 1 drop of 0.5% tropicamide and 1 drop of 2.5% phenylephrine, followed by a second drop of each 10 minutes later. Fluid supplementation (up to 10 mL/kg/h lactated Ringer’s Solution) was administered as a subcutaneous bolus after the procedure.

### FAM imaging

The Heidelberg Spectralis HRA+OCT imaging platform (Heidelberg Engineering) was used to collect FAM still images and video clips at a total sensitivity setting of 70–107 using a 55-degree lens at baseline and at multiple points after the HEA procedure and probe administration. Automatic real-time tracking (ART) mode was employed, with 100 images averaged. In vivo still images were collected with ART averaging and normalization turned on for most images, unless otherwise stated. Phantom (fluorescein solution) imaging was performed by video clips, with ART averaging, with and without normalization. Quantification of vitreal haze fluorescence intensity was based on the fluorescence level over the optic nerve head (ONH), which should have minimal tissue autofluorescence while containing activated probe.

### OCT

The Heidelberg Spectralis HRA+OCT imaging platform (Heidelberg Engineering) with a 30-degree lens was used to collect high-resolution, vertical and horizontal, single-line scans through the fovea, macular volume, and peripapillary circular B-scans at baseline and at multiple timepoints after the HEA procedure. RNFL thickness values were determined using Heidelberg proprietary software, with manual adjustment of the segmentation lines for better accuracy. RNFL thickness for Cy0407 was averaged on individual sectors of the ONH only using temporal superior (TS), nasal superior (NS), temporal inferior (TI), and nasal inferior (NI). For Cy0410 and Cy0302, we defined superior RNFL thickness at the ONH as the average of TS and NS, and inferior RNFL thickness at the ONH as the average of TI and NI.

### Visual electrophysiology

The LKC UTAS Electrodiagnostic Testing System with EMWin software (LKC Technologies, Inc., Gaithersburg, MD) was used to collect scotopic and photopic ffERG and photopic flash cortical VEP response waveforms from the retinas and visual pathways.

All animals were anesthetized as described above. We administered 1% tropicamide, 2.5% phenylephrine HCl, and proparacaine HCl ophthalmic solutions before visual electrophysiology. Active ERG-jet^™^ corneal contact lens electrodes were referenced to subdermal needle electrodes (disposable 29 G sterile surgical steel) that were situated at the ipsilateral outer canthi of both eyes. A subdermal needle electrode was inserted in the upper limb as the ground. Corneal contact lenses were applied to the ocular surfaces after 2.5% hypromellose ophthalmic solution had been applied to each recording contact lens. Four subdermal needle electrodes (2 active electrodes overlying the occipital cortices and 2 situated at the vertex) were inserted into the scalp for VEP response recordings. After the visual electrophysiology procedures, Optixcare (ocular lubricant) and/or antimicrobial combination ophthalmic ointment (e.g., neomycin and polymyxin B sulfates and bacitracin zinc) were applied to the corneal surfaces prophylactically and to maintain corneal surface hydration.

Following at least 1 h of dark adaptation, flashes of increasing strength were presented binocularly. The dark adaptation series flash strengths were -4.4, -3.2, -2.6, -2.2, -1.6, -1.2, -0.60, -0.20, and +0.40 log cd∙s∙m^-2^. ERGs were based on the average response to 5 flashes, presented with an inter-stimulus interval of 5 s. Following 10 min of light adaptation to 30 cd/m^-2^ white background in the ganzfeld bowl, ERGs were recorded at flash strengths of -2.2, -1.6, -1.2, -0.60, -0.20, +0.4, +0.6, and +1.20 log cd∙s∙m^-2^, with a 5-sec inter-stimulus interval. Following the light adaptation series, a 30.3-Hz flicker ERG was recorded, followed by 1.0-, 5.0-, and 10.0-Hz flash averages using a 4-channel montage to record right and left occiput VEPs from the right eye (oculus dexter [OD]) and subsequently from the left eye (oculus sinister [OS]).

### HEA procedure model of RGC injury

HEA was performed as previously described [19]. Briefly, prior to the HEA procedure, baseline FAM images and spectral domain OCT data were collected. Digital color fundus photography data were obtained using the TRC 50EX retinal camera (Topcon Corp., Tokyo, Japan) and captured using an EOS 5D Mark 2 camera (Canon, Tokyo, Japan) connected to the retinal camera.

All animals were anesthetized as described above. Pupils were dilated using topical 1% tropicamide and 2.5% phenylephrine HCl drops. The corneal surfaces were cleaned and sterilized using a 2.5% betadine solution, and any subsequent rinsing was performed using sterile balanced salt solution (BSS). The animal’s head was supported in a holding device (not a stereotaxic apparatus) to maintain a stable position. Proparacaine HCl (0.5% ophthalmic solution) was administered as a local anesthetic. A wire speculum was used to retract the eyelids. Two 25-gauge cannulae were inserted 4 mm posterior to the corneal limbus in the 2 o’clock and 10 o’clock meridians through the conjunctiva and sclera using trocars. A fiber optic light was passed through a 25-gauge cannula, and a sharp-tipped endodiathermy probe was passed through the other. A flat contact lens was placed on the cornea. Hydroxypropyl methylcellulose ophthalmic solution (2.5%) served as an optical couple. The retina was then visualized with a stereo operating microscope. Contiguous endodiathermy spots were placed along the inferior 180° adjacent to the optic nerve margin in the ODs of Cy0410 and Cy0302. The individual diathermy spots were created using enough energy to cause retinal whitening. Endodiathermy was not applied directly over the large retinal vessels. After the HEA procedure, the cannulae were removed; sutures were not required. Subconjunctival injections of an antibiotic (cefazolin, up to 25 mg/kg) and a corticosteroid (up to 20 mg of triamcinolone acetonide) were given at the end of the procedure. A mild analgesic (1.5 mg/kg flunixin meglumine IM) and an antibiotic (cefazolin, 25 mg/kg, IM) were also administered systemically on the same day as and for 2 days after the HEA procedure.

### Synthesis of cell-penetrating peptide probe TcapQ488 and dTcap488

Peptide TcapQ488 (Ac-rkkrrorrrGK-(QSY7)DEVDAPC(AF488)-NH2) was synthesized as described previously [10, 12, 15]. This activatable peptide probe consists of an all D-amino acid modified TAT cell–penetrating peptide sequence, an L-amino acid effector caspase recognition sequence (DEVD), a quencher (QSY7), and Alexa Fluor-488. Standard solid-phase N-α-Fmoc chemistry was used to synthesize the peptide (Tufts University Peptide Synthesis Core, Boston, MA). Thiol conjugation of Alexa Fluor 488 at the C-terminus enables intracellular accumulation of the fluorophore fragment after executioner caspase-mediated cleavage and loss of fluorescent quenching. Peptide Ac-rkkrrorrrgk-devdapc(AF488)-NH2 (dTcap488) is a variation of the TcapQ488 without quencher and consists of all D-amino acids; therefore, it is not cleavable and is constitutively fluorescent. Fluorophore Alexa Fluor-488 was chosen for compatibility with the clinically available Spectralis FAM imaging instrument. After conjugation, sterility (Charles River Endosafe PTS bacterial endotoxins test, USP <85> standards) and pyrogenicity (USP <71> standards) tests were performed on peptides to ensure safe use in the 3 cynomolgus macaques.

### Intravitreal peptide probe injection

All animals were anesthetized as described above. Baseline slit lamp examination was performed to ensure that the media was clear and that the retina and optic disc were normal. Intraocular pressure was measured to confirm that it was within normal range. One drop each of 0.5% tropicamide and 2.5% phenylephrine were administered, followed by a second drop of each 10 minutes later. The injection site was sterilized using standard procedures for ocular surgery with ophthalmic povidone-iodine and then rinsed with sterile BSS. The TcapQ488 or dTcap488 probes were diluted in ~30 μl of BSS. These probe stock concentrations were further mixed with BSS to achieve injectates containing 3, 6, or 12 nmol TcapQ488 or 6 nmol dTcap488, which were injected into the monkey vitreous. IM meloxicam (0.2 mg/kg) and buprenorphine (if needed) were administered for analgesia. Animals were observed for 2- or 3-days after the procedure and administered IM meloxicam (0.1 mg/kg) once daily as needed.

## Results

We performed baseline studies in both eyes in all 3 monkeys (Cy0407, Cy0410, and Cy0302), including ffERG and VEP tests (S1 Fig), slit lamp examination, intraocular pressure measurement, and Heidelberg Engineering Spectralis scanning, which includes FAM and OCT imaging.

### Dose-Limiting Toxicity (DLT) in healthy retinas

**Fig 1:**
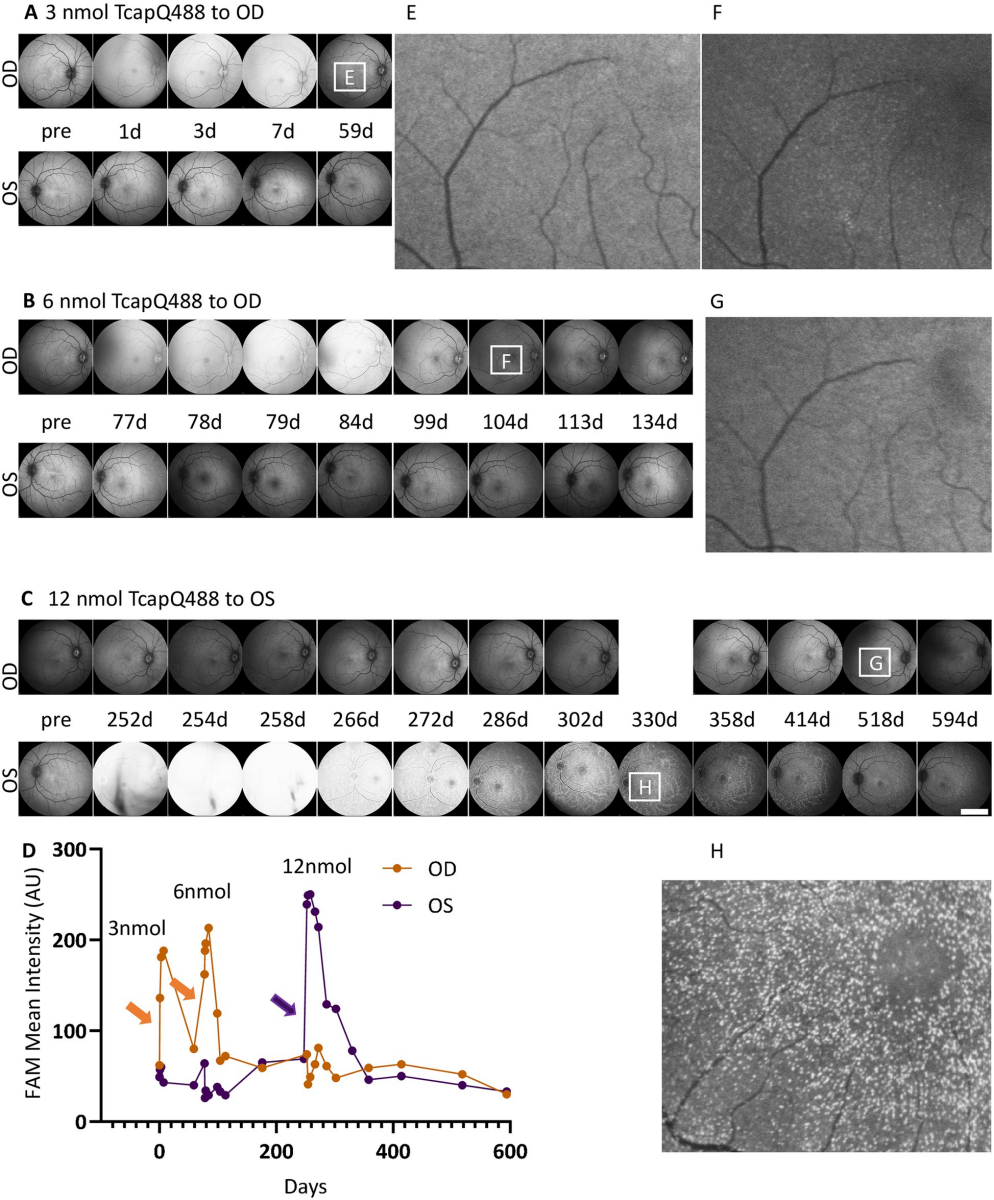
TcapQ488 dose testing and dose-limiting toxicity in healthy retinas of Cy0407. Numbers between images were in chronological order of days post 3 nmol probe injection to OD. The images following the pre-images in A-C were always 1 day post the probe injection at different doses to OD or OS, respectively. All total sensitivities of images were set at 101 ± 2. **A**) 3 nmol TcapQ488 intravitreal injection to OD led to long lasting haze in the vitreous with no detectable autoactivation (also **D**, **E**), while intravitreal injection of 6 and 12 nmol TcapQ488 to OD and OS, respectively, led to haze in the vitreous (**B**, **C, D**), and TcapQ488 autoactivation at GCL detected by Spectralis FAM imaging (**F, G** and **H**). Arrows in **D** indicated the time of probe injections. Fluorescence angiography mode (FAM), Oculus Dexter (OD), Oculus Sinister (OS), Ganglion cell layer (GCL). Scale bar (5 mm).

**Fig 2:**
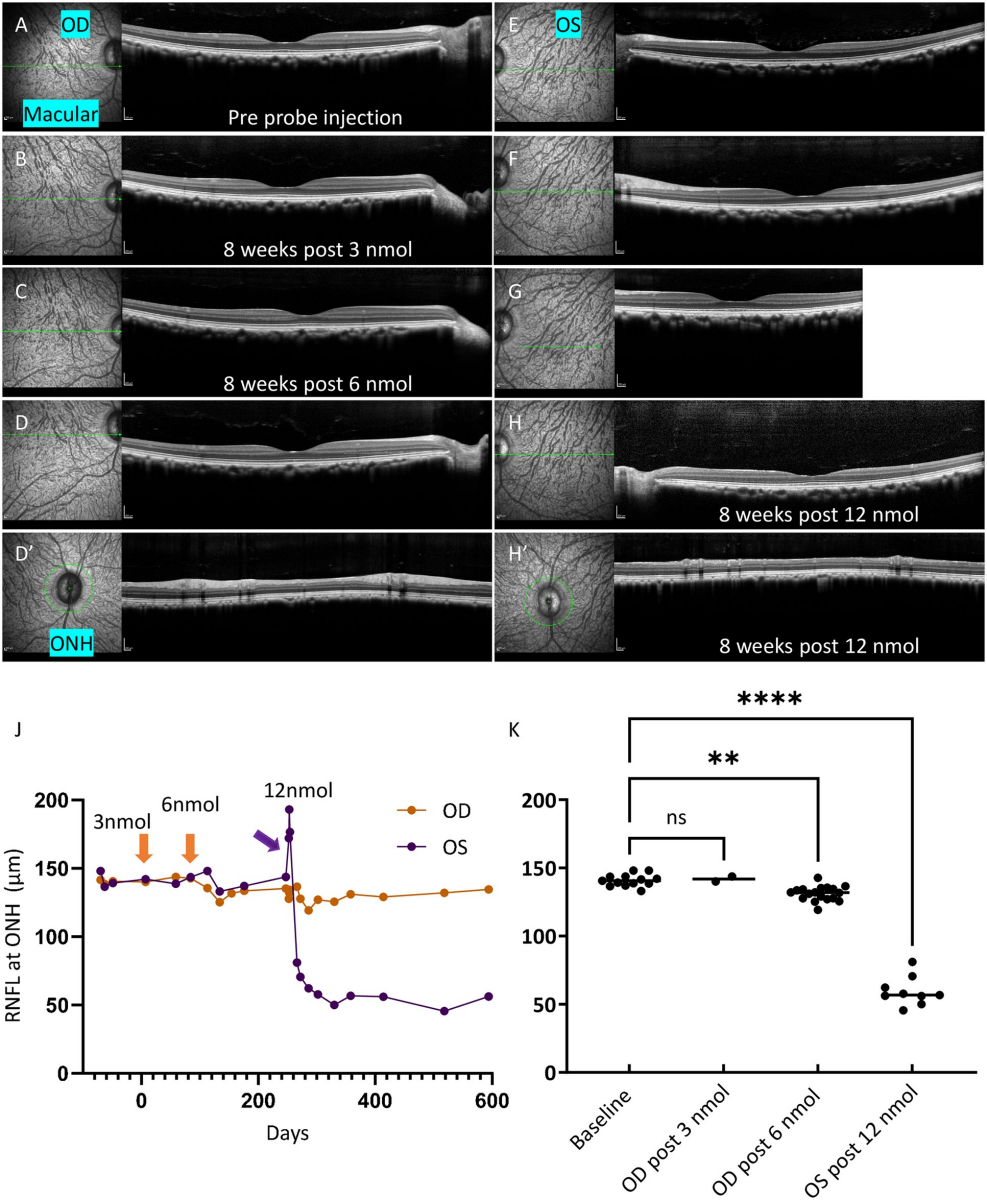
Measuring changes in RNFL thickness by OCT yields 3 nmol as an optimal dose. OCT scans through the macula in OD (**A**-**D**) and OS (**E**-**H**), and OCT scans at ONH in OD (**D’**) and OS (**H’**) of Cy0407. Horizontal arrows in infrared reflectance images (**A**-**D** and **E**-**H**) on the left indicated the single line scan position for the OCT images showing on the right, respectively. Green circle in Infrared reflectance images (**D’** and **H’**) indicated peripapillary circular B-scan postition for the OCT images showing on the right, respectively. **A** and **E** were baseline images for OD and OS of Cy0407, respectively, prior to TcapQ488 injection. **B** and **F** were acquired images at 8 weeks post 3 nmol TcapQ488 injection to OD, and balanced salt solution injection to OS, respectively. **C** and **G** were acquired images at 8 weeks post 6 nmol TcapQ488 to OD,. **D, D’, H** and **H’** were acquired images at 8 weeks post 12nmol TcapQ488 to OS. RNFL thickness, averaged from temporal superior, temporal inferior, nasal superior and nasal inferior of the retina, were quantified at ONH through out the experiments in both eyes (**J, K**). Arrows in **J** indicated the time of probe injections. Intravitreal injection of 12 nmol TcapQ488 to the OS led to transient edema followed by RNFL thinning in the retina (**J**). Optical coherence tomography (OCT), Oculus Dexter (OD), Oculus Sinister (OS), Optic nerve head (ONH), retinal nerve fiber layer (RNFL), Scale bar (200 μm). ** p = 0.0047, **** p<0.0001.

As a first study in NHP, determination of a non-toxic working dose of the probe was the initial priority (Figs 1 and 2). Based on cell-based assays [9, 10, 14] and estimated intravitreal concentration, we intravitreally injected 3 nmol TcapQ488 into the healthy OD and BSS into the healthy OS of Cy0407 (Fig 1A and 1D). Fig 1A and 1D show a transient fluorescent signal (haze) detected from the OD vitreous by Spectralis FAM scanning; the fluorescent haze intensity in the vitreous increased at days 3 and 7, and resolved by day 59. However, there was no TcapQ488 activation detected in the retina in the GCL after the fluorescent haze returned to baseline (< 1 standard deviation of baseline [Fig 1A, OD, 59d]). Furthermore, the macular volume and thickness values derived from the OCT scans were comparable to the pre-probe injection baseline (Fig 2A and 2B), and the RNFL thickness was comparable to the baseline around the ONH (Fig 2J and 2K) at 8 weeks after the 3 nmol TcapQ488 injection.

To identify a potential dose limiting toxicity in healthy retina, we intravitreally injected an additional 6 nmol TcapQ488 into the OD of Cy0407 (Fig 1B and 1D). The haze from the injection lasted approximately 3 weeks. FAM scanning detected TcapQ488 activation (Fig 1B and 1F) in the GCL after the haze had cleared (Fig 1B, OD, 104d). Furthermore, the peripapillary RNFL thickness around the ONH (average of TS, NS, TI, and NI) decreased to 131 ± 1 μm (SEM), a significant difference when compared with both baseline (140 ± 1 μm [SEM]) and post 3 nmol TcapQ488 injection (141 ± 1 μm [SEM]) (Fig 2J and 2K). Overall, the data here indicated that consecutive 3 and 6 nmol TcapQ488 intravitreal injections in a healthy cynomolgus eye triggered apoptotic death in the GCL, activated TcapQ488 (autoactivation), and caused RGC axon loss. The optical probe dose limit may have been reached.

To confirm, intravitreal injection of 12 nmol TcapQ488 into the healthy OS caused extensive probe autoactivation in the GCL (Fig 1C and 1D). The haze from the injection lasted approximately 12 weeks. At 3 days post 12 nmol injection (Fig 1C, OS, 254d), we also verified the haze detection at various instrument total sensitivity settings, testing as low as 70 (S2 Fig). The punctate-labeling pattern in the OS retina at 12 weeks after probe injection (Fig 1C, OS, 330d) resembled the NMDA model of RGC injury in rodents detected by the same TcapQ488 probe [15]. TcapQ488 (12 nmol), mimicked the NMDA excitotoxicity model, caused synchronized RGC cell body injury and activation of the peptide probe, consistent with caspase activation. Furthermore, OCT peripapillary circular scans at the ONH detected RNFL thinning, consistent with RGC death and axon loss in the OS (Fig 2H and 2H’). The peripapillary RNFL average thickness measured longitudinally showed that the 12 nmol probe injection initially induced thickening of the RNFL, followed by sharp thinning of the RNFL, with a mean difference of -81 ± 3 μm (SEM) compared to baseline by approximately 2–3 weeks (Fig 2J and 2K).

### Spectralis FAM imaging normalization function

Spectralis FAM imaging acquires sharp-contrast fluorescent images when the normalization function mode is turned on. To better understand the effect of the normalization function on FAM imaging, especially for intensity estimation of the fluorescent “haze” shown in Fig 1, we performed further investigation of a fluorescent phantom and additional in vivo imaging, with and without the normalization function. FAM imaging total sensitivity (gain) was set at 70, 80, and 90 when acquired using autofluorescence video clips of a fluorescein solution of fixed concentration (phantom, n = 3) (S3A, S3B, and S3G Fig). When the normalization function was turned off, both the background and phantom signal intensities showed a linear relationship with the sensitivities. When the normalization function was on, the background signal intensity was set to approximately 7% ± 0.2%, and the phantom signal intensity was increased to approximately 68% ± 3% of the maxima brightness, regardless of the total sensitivity setting (S3G Fig).

For in vivo imaging, FAM imaging sensitivity was set at 80, 90, 100, and 107. The vitreous fluorescent intensity was imaged from the OD of Cy0410 before any procedure (background) and after the HEA procedure plus TcapQ488 intravitreal injection, with and without the instrument normalization function. When the normalization function was off, fluorescent intensity was acquired at the vitreous before or after the HEA procedure plus probe injection, and both showed a linear relationship with the sensitivity settings (S3C, S3E, S3H, and S3J Fig). When the normalization function was on, before the procedure, normalization clamped the intensities close to the normalization off value, around the mid-sensitivity range (S3H Fig). This is consistent with the finding from the phantom (S3G Fig). However, after the HEA procedure plus probe injection, the signal intensities showed a linear relationship with the total sensitivities (S3J Fig). Therefore, although the normalization function was turned on for all of the FAM acquisitions shown in Fig 1, S3 Fig data indicated that the quantification for vitreous haze fluorescence intensity from the in vivo images represented true relative signal intensities.

### Intravitreal TcapQ488 injection revealed axonal injury in the HEA model of RGC axonal injury

**Fig 3:**
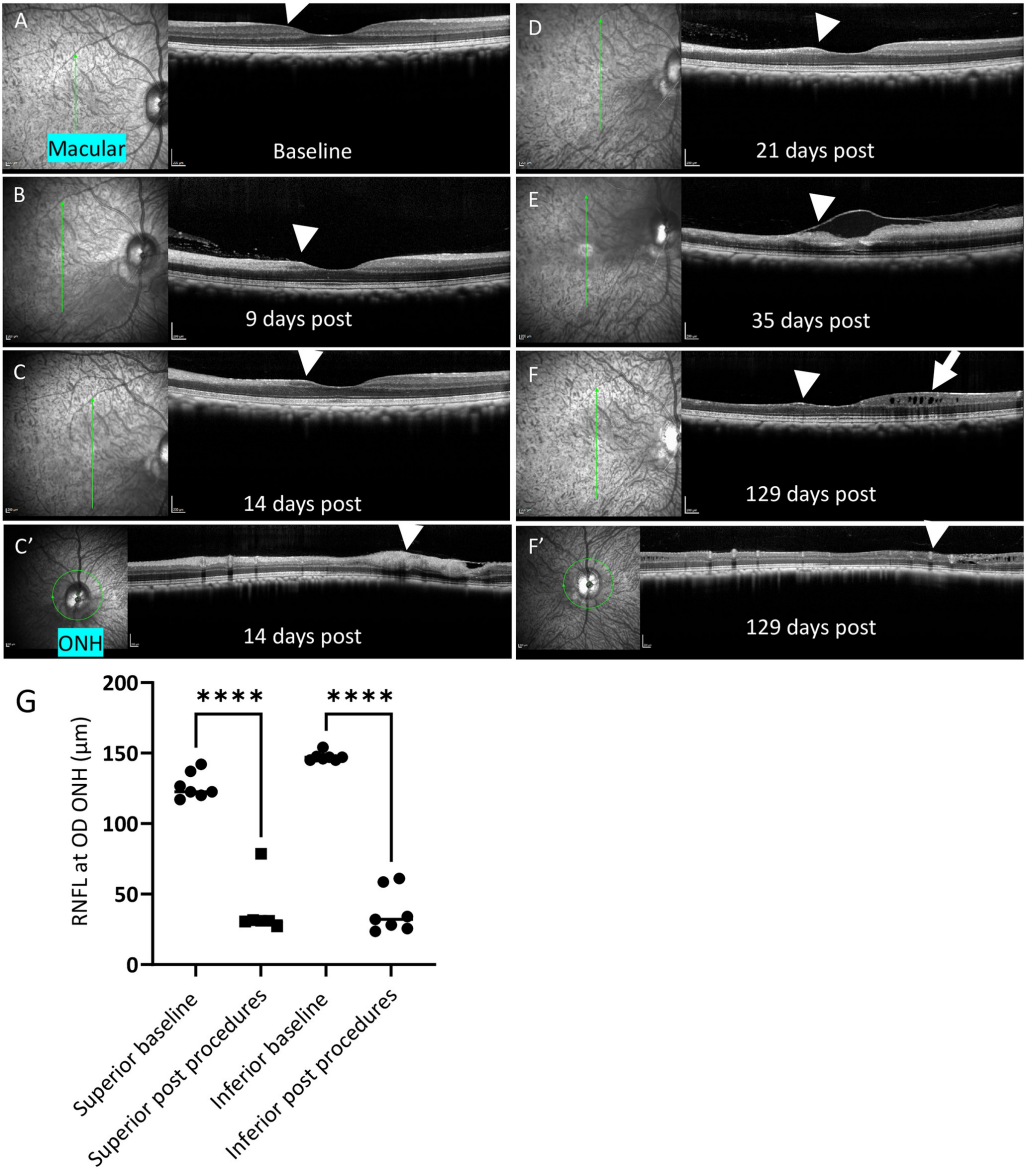
HEA and high dose probe injection caused thinning of RNFL (Cy0410). OCT scans through the macula in OD (**A**-**C** and **D**-**F**) and OCT scans at the ONH in the OD (**C’** and **F’**). Vertical arrows in infrared reflectance images (**A**-**C** and **D**-**F**) on the left indicated the single line scan position for the OCT images showing on the right, respectively. Green circle in Infrared reflectance images (**C’** and **F’**) indicated peripapillary circular B-scan position for the OCT images showing on the right. **A**) baseline image for Cy0410 prior to HEA. **B**) 9 days post HEA procedure and 2 days post 3nmol TcapQ488 injection. **C** and **C’**) 14 days post HEA procedure and 7 days post 3nmol TcapQ488 injection. **D**) 21 days post HEA procedure and 14 days post 3nmol TcapQ488 injection. **E**) 35 days post HEA procedure and 1 day post additional 6 nmol TcapQ488 injection. **F** and **F’**) 129 days post HEA procedure. White arrowheads in OCT images pointed toward inferior retina. Before HEA procedure, inferior retina immediate to the fovea showed clear layers of RNFL and other layers (**A**). In contrast, marked thinning of the inferior RNFL and GCL at the macular were observed after 9 days post HEA procedure (**B**). White arrow in OCT image in **F** pointed toward microcystoid macular degeneration in inner nuclear layer of superior retina post HEA and sequential 3, 6 nmol probe injection. **G**) RNFL thickness at superior (averaged from temporal superior and nasal superior), and at inferior (averaged from temporal inferior and nasal inferior) were quantified at ONH through out the experiments. OD inferior RNFL was significantly thinner after HEA and probe application compared to baseline condition. OD superior RNFL also went thinning due to the additional 6 nmol probe injection led toxicity. Optic nerve head (ONH), Retinal Nerve Fiber Layer (RNFL), Ganglion Cell Layer (GCL). Scale bar (200 μm). ****, P<0.0001.

**Fig 4:**
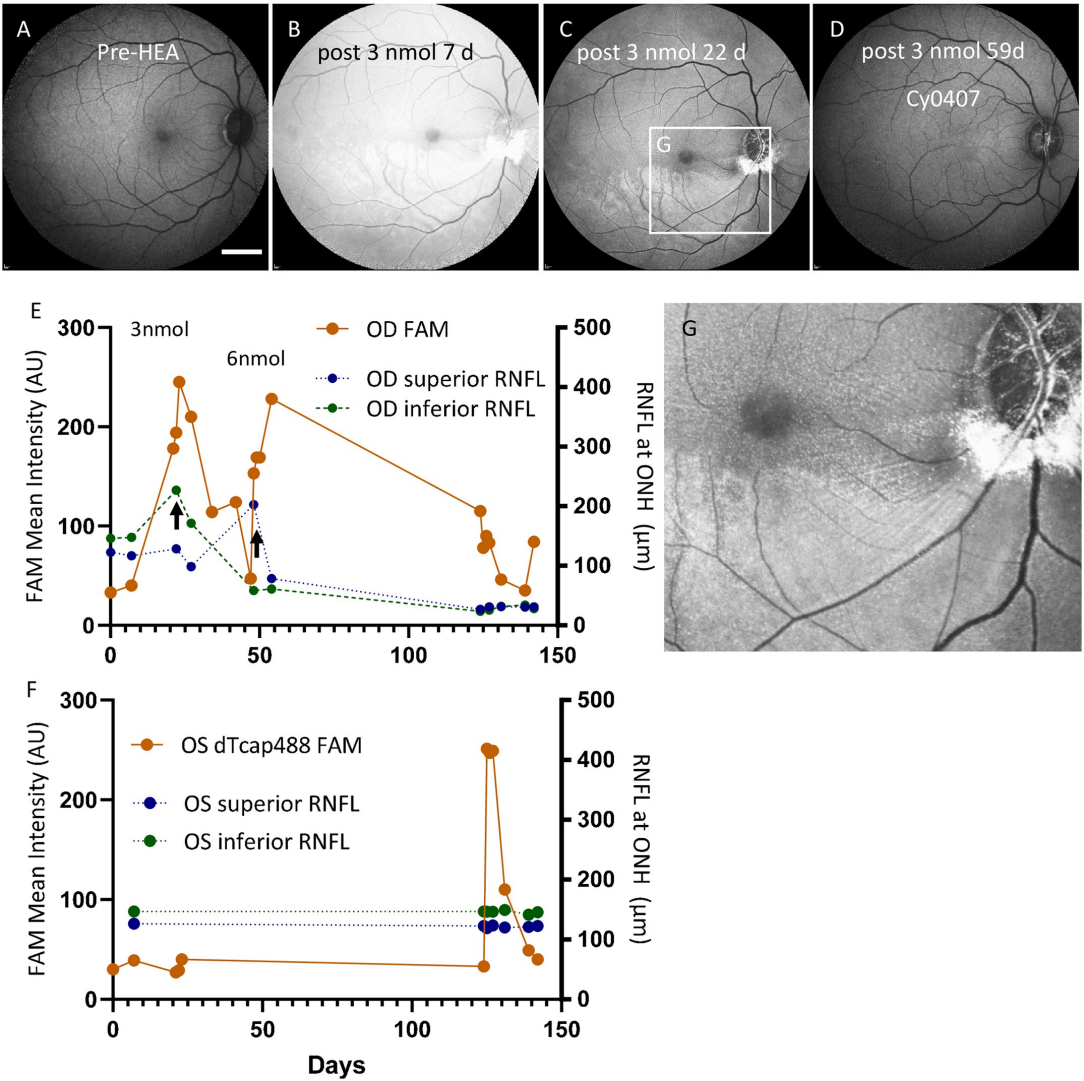
TcapQ488 detected HEA-induced RGC injury in vivo by Spectralis FAM imaging corresponding to RNFL thinning in Cy0410. **A**) baseline image for Cy0410 prior to HEA in OD. **B**) at 7 days, haze of intravitreally injected 3 nmol TcapQ488 obscured the signal from HEA treated OD retina. **C**) at 22 days post 3 nmol TcapQ488 injection, activated TcapQ488 signal was detected from HEA treated inferior retina of Cy0410. They were punctate fluorescent signals found in GCL, fluorescently labeled HEA sites and fluorescent streaks radiating from the HEA sites. **D**) As a control, intravitreal injection of 3 nmol TcapQ488 in healthy OD of Cy0407 did not lead to probe activation in the retina. **E)** In Cy0410 OD, consecutive intravitreal 3 and 6 nmol TcapQ488 injections caused long lasting haze in the vitreous showing in orange curve. Averaged TS and NS (blue dotted line), TI and NI (green dotted line) showed RNFL thickness at ONH changes along the experiments. Black arrows indicate the time of 3 nmol and 6 nmol injections. **F**) In Cy0410 OS, haze from non-cleavable, non-quenched dTcap488 (6 nmol) in the vitreous of the healthy OS lasted approximate 2 weeks as shown in the orange curve. Averaged TS and NS (blue dotted line), TI and NI (green dotted line) in the OS before and after the dTcap488 injection both showed no sign of change in RNFL thickness. Fluorescence angiography mode imaging (FAM), Right eye (OD), Left eye (OS), Ganglion Cell Layer (GCL), Hemiretinal Endodiathermy Axotomy (HEA), Retinal nerve fiber layer (RNFL), Temporal Superior (TS), Temporal Inferior (TI), Nasal Superior (NS), Nasal Inferior (NI), Scale bar (2 mm).

To verify that TcapQ488 detected caspase-activated neuronal cell death in our model of RGC axonal injury in NHPs, we placed HEA sites along the inferior 180° adjacent to the optic nerve margin in the OD of a second cynomolgus macaque, Cy0410, followed by TcapQ488 intravitreal injections (Figs 3 and 4).

We monitored RNFL thinning caused by the HEA procedure in the OD via Spectralis OCT imaging. Fig 3 shows longitudinal RNFL thickness change at the macula before and after the HEA procedure. Representative OCT images show RNFL thinning at 9 days after the HEA procedure (temporally 2 days after 3 nmol TcapQ488 intravitreal injection, Fig 3B), and at 14 days (Fig 3C), and at 21 days (Fig 3D) post procedure. Qualitatively, inner retinal layers at the inferior retina were smeared and indistinct, while those at the superior retina retained clear boundaries. The success of the HEA model was also confirmed via marked thinning of the peripapillary RNFL (average of TI and NI, Fig 3C’, 3F’ and 3G) in the inferior retinas as measured by OCT, for a mean difference from baseline of -87 ± 2 μm. In addition, we observed inner limiting membrane detachment at the macula and edema in the superior retina at 35 days after the HEA procedure and 1 day after an additional 6 nmol TcapQ488 intravitreal injection (Fig 3E). At 129 days after the HEA procedure, a representative OCT image (Fig 3F) confirmed RNFL thinning in the inferior retina and microcystoid macular degeneration in the superior macula.

TcapQ488 (3 nmol) was injected into the OD vitreous 1 week after HEA (Fig 4). At 7 days after probe injection, haze still obscured the retinal signal (Fig 4B). Once the haze returned to background (22 days after the 3 nmol TcapQ488 intravitreal injection), 3 qualitative fluorescent signatures were observed in the inferior retina (Fig 4C and 4G): densely packed punctate fluorescent signals, thin fluorescent streaks radiating from the HEA sites, and a strong patch of fluorescent signal from the HEA sites. At the same time, labelling of cell bodies was seen in the superior parafoveal area as expected due to involvement of the papillomacular bundle with endothermy lesions placed along the inferotemporal-temporal peripapillary region. In contrast, as a control, 3 nmol TcapQ488 was intravitreally injected in the healthy eye of the first NHP, Cy0407, and did not result in a fluorescent signal in the retina (from the beginning (Fig 1A) out to 8 weeks (Fig 4D)).

After the initial 3 nmol injection, an additional 6 nmol TcapQ488 was injected into the OD vitreous at 4 weeks to compare consecutive probe injections within the healthy eye of Cy0407 (Fig 4E and S4 Fig). Note that the haze in the vitreous from the probe cleared in approximately 3 and 12 weeks for the 3 and 6 nmol intravitreal TcapQ488 injections, respectively (Fig 4E). Thus, after the initial 3 nmol injection, high quality signals were observed. However, an additional 6 nmol TcapQ488 intravitreal injection was toxic, as evidenced by reporter autoactivation in the GCL (S4A–S4G Fig), as well as RNFL thinning in the superior retina at a mean difference from baseline of -88 ± 8 μm (Fig 3G). In addition, RNFL thinning further extended in the inferior retina at a mean difference from baseline of -109 ± 6 μm (Fig 3G).

### dTcap488 monitored probe delivery and axonal transport in healthy eye

To further study RGC uptake and transport kinetics of our peptide probe in NHP retina, we intravitreally injected a non-quenched, non-cleavable control probe (dTcap488 6 nmol) into the healthy OS of Cy0410 to monitor probe delivery, RGC uptake, and axonal transport via its constitutive fluorescent signal. Non-quenched dTcap488 at 6 nmol emitted a much stronger, longer lasting peak fluorescent intensity in the vitreous (S4 Fig). The ONH was also better visualized with higher contrast in the retina at the non-saturated acquisition setting, consistent with the probe being endocytosed and actively transported by RGCs, traveling down their axons, and entering the ONH (S4I’ Fig). In healthy NHP eyes, it took approximately 2 weeks for the haze in the vitreous to clear (Fig 4F and S4 Fig). We expected to see fluorescent signal accumulation either at the cell nuclei, cell bodies, or axons when the vitreous had cleared. Surprisingly, however, there was no detectable probe fluorescent signal at the retina or ONH on Spectralis FAM imaging 2 weeks after probe injection, when the haze had cleared. These results suggested that RGC uptake and subsequent axonal transport of dTcap488 effectively cleared the vitreous and carried the probe away from the retina into the optic nerve.

### Intravitreal TcapQ488 injection prior to the HEA procedure led to minimal probe labeling in GCL

**Fig 5:**
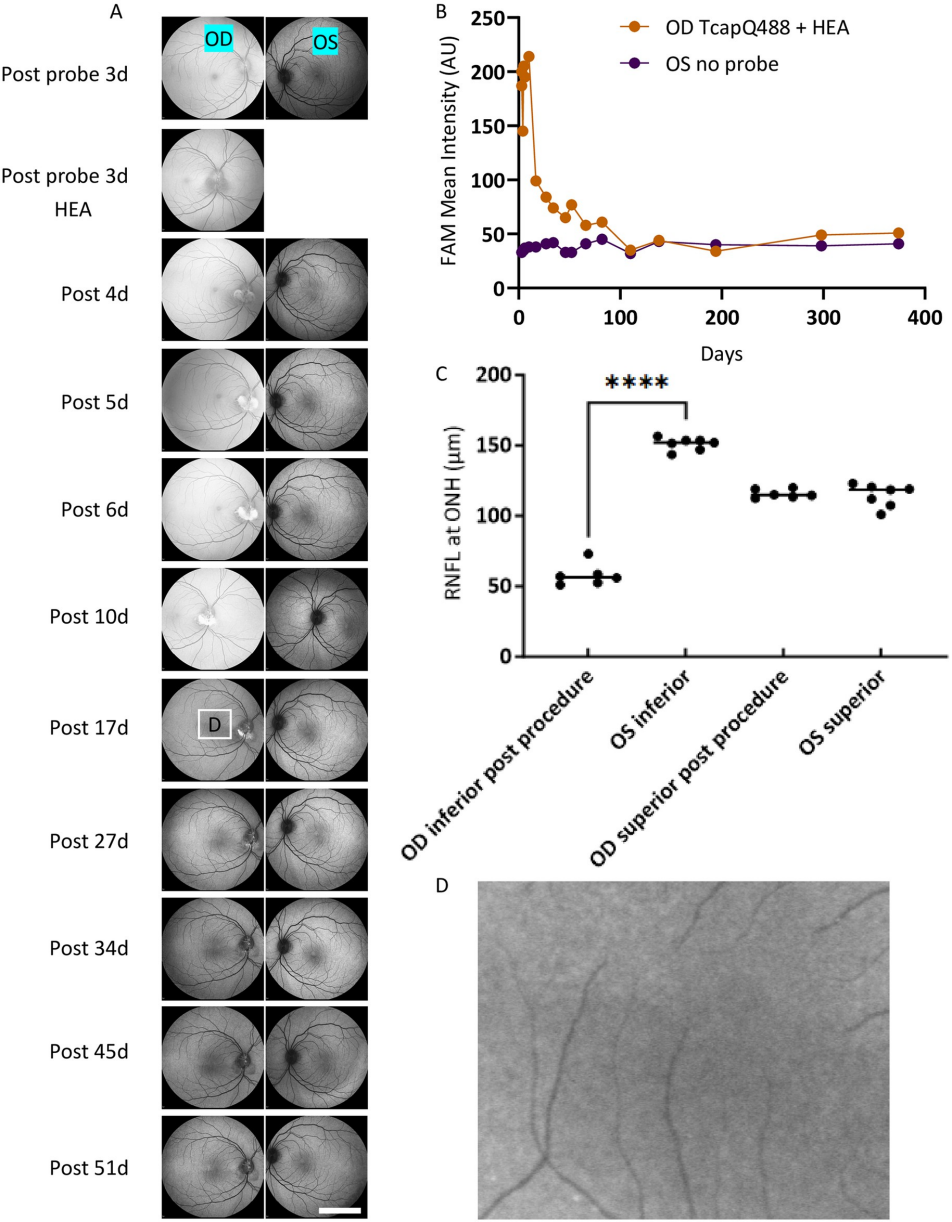
TcapQ488 injection followed by HEA procedure in OD of Cy0302 showed inferior image quality and refined visualization compared to post HEA injection of TcapQ488. All total sensitivities of the images were set at 100. **A**) Numbers by the images were timeline days post probe injection to OD. OD had intravitreal TcapQ488 injection followed by HEA procedure 3 days later. The TcapQ488 haze in the vitreous lasted approximate 14 days. Activated TcapQ488 was detected at the HEA site but not at other parts of the retina, including the inferior site of the retina. OS eye had no probe injection nor other procedure. **B**) Fluorescent haze was detected from OD vitreous. **C**) RNFL thickness at ONH was quantified as average of sectors Temporal Inferior and Nasal Inferior, or average of sectors Temporal Superior and Nasal Superior for inferior or superior retina, respectively. RNFL thickness at OD inferior was significantly thinner than the OS inferior due to the HEA procedure. Fluorescence angiography mode (FAM), Retinal nerve fiber layer (RNFL), Optic nerve head (ONH). Scale bar (5 mm). ****, P<0.0001.

To further confirm that TcapQ488 pre-loaded into the eye could not detect later RGC injury because rapid cell uptake and axonal transport of the probe led to reduced availability of the probe at the moment of injury, we pre-injected 6 nmol TcapQ488 into the healthy vitreous of NHP Cy0302. The HEA procedure was performed 3 days later. Consistent with the rapid axonal transport of the probe in healthy RGCs, the majority of the probe was transported away from the retina by the time the injury occurred; only a few punctate TcapQ488 fluorescent signals were detected in the pre-loaded retina, even at 6 weeks after the HEA procedure (Fig 5A). Haze in the vitreous was cleared in 2 weeks after probe injection (Fig 5A and 5B). Note the striking difference in the temporal relationship of probe injection and appearance of the fluorescent patch over the HEA sites. Prior to HEA, the fluorescent intensity inferior to ONH remained low (Fig 5A, OD, 3 days after probe delivery), indicating that axonal transport delivered the intact probe into the optic nerve. However, after HEA, the remaining probe gradually increased in signal at the HEA sites (Fig 5A, OD, post 4d-10d), as probe was no longer transported and was cleaved at the HEA sites. Reduced inferior RNFL thickness caused by the HEA procedure was confirmed by Spectralis OCT in the Cy0302 OD (Fig 5C, mean difference of -93 ± 3 μm between the OD and OS inferior retina (average of TI and NI)). Spectralis FAM imaging also confirmed that the HEA procedure did not form characteristic fluorescent signals of scar tissue during the study (Fig 5A, OD, Post probe 3d HEA).

## Discussion

In this study, we estimated the DLT of intravitreal injections of TcapQ488 probes in NHPs and characterized early signals of efficacy. Both were successfully identified, but we also detected an emergent property of prolonged probe-induced fluorescence haze in the vitreous of NHPs not observed in prior rodent models. The quenching efficiencies of the single quencher-fluorophore pair of the TcapQ488 probe were previously estimated at between 92% and 99% [10]. Therefore, even the native quenched probe could contribute a transient fluorescent haze from an inevitably unquenched probe fraction in the vitreous, obscuring the view of the retina on Spectralis FAM imaging (n = 3). It would appear in this scenario that the path length from the laser entrance to the retina and therefore the out-of-plane residual fluorescence does not scale from rodents to NHPs. When the vitreal haze cleared, we observed fluorescence activation of the probe over baseline retinal autofluorescence in the HEA model of RGC degeneration including cell body activation consistent with rodent models [11, 12, 15]. No evidence of diffuse retinal toxicity or damage to the visual pathways was detected in the three NHPs studied, although discrete lesions might not be detected using ffERG and VEP.

Interestingly, the duration of the transient haze corresponded inversely to the health of the retinas (n = 3). In Cy0407, we estimated that it took approximately 2 and 3 weeks for healthy OD to clear the consecutive injections of 3 and 6 nmol TcapQ488 from the vitreous, respectively. In the healthy OS of Cy0410, 6 nmol dTcap488 from the vitreous cleared in about 2 weeks. Furthermore, preloaded 6 nmol TcapQ488 injected into OD of Cy0302 were cleared in approximately 2 weeks. In comparison, in Cy0410, the HEA-injured OD prolonged the duration to 4 and 12 weeks for 3 and 6 nmol TcapQ488, respectively. These data suggested that with RGC injury, an apparent correlative decrease in axonal transport kinetics occurred in NHPs, thus slowing down the clearance of the vitreal haze while simultaneously enhancing the signal of cleaved probe retained in RGC cell bodies. Moreover, dTcap594, effectively an optically-shifted dTcap488 probe composed only of the modified TAT peptide sequence conjugated to Alexa Fluor 594, has been reported to label the RGC axon and optic nerve in rats [11]. Our results in NHPs are consistent with those in rats, indicating that the modified TAT sequence served as an axonal transport signal, which engaged the axonal machinery in healthy RGCs in NHPs to move TcapQ488 away from the vitreous via axonal transport. Our overall results here were consistent with a model in which TcapQ488 is rapidly endocytosed into healthy primate RGCs and then transported anterograde down the axons to leave the eye; such transport and the apparent rate constants observed represent a significant contributor to clearance of the probe from the eye.

Because the physiology of NHPs is closely related to humans, we estimate that the 2–12 weeks required for washout of the vitreal haze in NHPs may apply to humans as well. Thus, in practical terms, returning ophthalmologist office visits after 2 weeks or more would be required to complete the diagnostic assessment, which may be inconvenient and cost-preventative. Additional drawbacks to the use of the procedure as demonstrated herein may be blurry vision caused by lingering fluorescent probe in the vitreous and general hesitation for using intraocular injection procedures for diagnostics purposes only. Therefore, another probe or alternative application route should be explored to address the haze issue in the vitreous exposed on scale-up from rodent to NHP models.

We estimated intravitreal doses of 12 nmol TcapQ488 as the DLT dose for use in detection of caspase-mediated apoptotic RGC death in NHPs on the basis of FAM and OCT imaging. Conversely, intravitreal injection of 3 nmol TcapQ488 in healthy OD of Cy0407 did not result in a detectable auto-activation signal in GCL (Fig 1A) or a detectable RNFL thickness change (Fig 2). The same applied to a 6 nmol dTcap488 intravitreal injection in the healthy OS of Cy0410 (Fig 4F). On the other hand, a 12 nmol TcapQ488 intravitreal injection clearly caused RNFL thinning and probe auto-activation in GCL of the OS of Cy0407 (Figs 1C and 2J, 2K), while an additional 6 nmol TcapQ488, delivered sequentially after 3 nmol, caused RNFL thinning and probe auto-activation in GCL in both healthy and HEA-treated eyes (Figs 2K and 4E, 4F). If the technology were to became available as a diagnostic, additional repeat dose testing and dose optimization would be required before final translation to match appropriate safety with instrument response and predictive value.

Most importantly, there was proof-of-principle evidence of probe activation in a NHP model of RGC degeneration. Intravitreal injection of 3 nmol TcapQ488 in the HEA-treated eye showed a distinct Alexa Fluor-488 fluorescent signal from cell bodies in GCL (Fig 4C). The results of our prior studies in rodents indicate that caspase activation colocalizes with activatable peptide probe in RGC cytoplasm [8, 12]. Our NHP results also support the notion that activated caspase-cleaved TcapQ488 and the released Alexa Fluor-488-peptide fragment reside in RGC cytoplasm. Indeed, free Alexa Fluor-488-fragments emitted a fluorescent signal within the RGC cytoplasm resulting in a punctuated retina as detected by Spectralis FAM imaging (Fig 4C and 4G). Furthermore, results from the TcapQ488 and dTcap488 experiments in NHPs suggested that axonal transport rapidly carried the probe out of the retina towards the brain after RGC cell body-mediated endocytosis. However, while there was evidence of activation of TcapQ488 without toxicity when NHPs were intravitreally injected with 3 nmol TcapQ488, the prolonged clearance time of the vitreous humor observed in NHPs likely precludes clinical translation with the current suite of ophthalmological fluorescent agents. Experiments exploring probe modification and delivery alternatives are underway.

## Supporting information

S1 Fig Full-Field Electroretinogram (ffERG) did not detect significant changes in retinal or retino-cortical function over the course of the studies. Representative ffERG data acquired under dark adaptation (scotopic series) and light adaptation (photopic series), Oscillatory potentials (OPs) data, 30.3-Hz flicker ERG amplitude and timing data and 5.0-Hz flash visual evoked potentials (FVEP) amplitude data from NHP Cy0407 are presented. Rod-mediated and cone-mediated A- and B-waves exhibited normal increases with flash strength, consistent with preserved outer (photoreceptors) and inner (bipolar cells; Müller cells) function. Generally, lower amplitudes were elicited following the first test, but scotopic and photopic ERG amplitudes remained relatively constant for the remainder of the study for all 3 animals. No instances of ‘flat’ or extinguished ERGs were noted. OPs amplitude, 30-Hz flicker ERG amplitude and timing and FVEP amplitude were unaltered across the course of the studies. No clear or consistent evidence of physiologically significant changes in retinal or retino-cortical function were evident in any of the 3 NHPs tested.
(PDF)

S2 Fig Spectralis FAM imaging at 3 days post 12 nmol TcapQ488 to OS of Cy0407 (also see image in Fig 1C OS 254d) at varies total sensitivity setting confirmed fluorescence haze in the vitreous. Numbers 100, 90, 80 and 70 were total sensitivity settings for each image below the number, respectively. The haze from 12 nmol TcapQ488 injection was confirmed by all total sensitivity settings from the vitreous obscuring fluorescence signals from GCL. Fluorescence angiography mode (FAM), Oculus Sinister (OS), Ganglion cell layer (GCL), Scale bar (2 mm).
(PDF)

S3 Fig Spectralis FAM imaging on varied total sensitivity settings, with/without normalization function on phantom fluorescent probe (A, B) and on Cy0410 retina before (C, D) and after (E, F) HEA/3 nmol TcapQ488 intravitreal injection procedures. Numbers in images are total sensitivity setting for the image when acquired, respectively. When Spectralis normalization function was turned off, fluorescent signal intensity detected from the phantom and its background corresponded with the total sensitivity setting, i.e., higher total sensitivity detected higher background intensity and fluorescent signal intensity (background signal subtracted) (A, G). When Spectralis normalization function was turned on, both background intensity and phantom fluorescent signal intensity were clamped at respective system defined signal and background optimal intensity regardless of the total sensitivity settings (B, G). During in vivo FAM imaging in the eyes of Cy0410 prior procedures HEA/intravitreal injection of 3 nmol TcapQ488, when Spectralis normalization function was turned off, detected FAM signal intensity (at ONH) corresponded with the total sensitivity settings (C, H). When Spectralis normalization function was turned on, FAM signal intensity at ONH was clamped at a level system defined as background signal (D, H). However, 3 nmol TcapQ488 intravitreal injection caused strong fluorescent haze in the vitreous, detected FAM signal intensity corresponded with the total sensitivity settings even the normalization function was turned on (E, F, J). Fluorescence angiography mode (FAM), Hemiretinal Endodiathermy Axotomy (HEA), Optic Nerve Head (ONH). (PDF)

S4 Fig Intravitreal injection of dTcap488 (6 nmol) to OS of Cy0410 resulted in significant multi-day low frequency background signal, and few foci of autoactivation. Longitudinal Spectralis FAM images of Cy0410 OD (**A-G**) and OS (**H-N**) were paired based on time of non-cleavable, non-quenched 6 nmol TAT peptide probe dTcap488 intravitreal injection. Punctate fluorescent signals detected at OD were from previous HEA procedure and consecutive TcapQ488 intravitreal injections (**A-G**) to OD. **I’**) one day post the probe injection when total sensitivity setting was at 61 (all other images were set at 100). Arrow pointed hyperfluorescent signal at ONH, suggesting dTcap488 was actively transported towards the brain via RGC axons. The dTcap488 haze stayed in the vitreous for approximately 15 days before being cleared out. At Post 15 days (M) and 18 days (N) post dTcap488 injection, there was few detectable fluorescent signals at GCL, and the ONH was fluorescently hypo labeled. Scale bar (2 mm).
(PDF)

S1 File Full description of NHP housing at WNPRC. (DOCX)
