# Supplementary figures and images for: In vivo scanning laser fundus and high-resolution OCT imaging of retinal ganglion cell injury in a non-human primate model with an activatable fluorescent-labeled TAT peptide probe
Source: PLoS One. 2024 Dec 6;19(12):e0313579. doi: 10.1371/journal.pone.0313579 (PMC11623487; doi:10.1371/journal.pone.0313579)

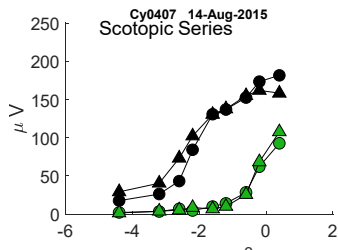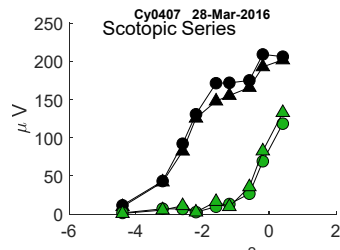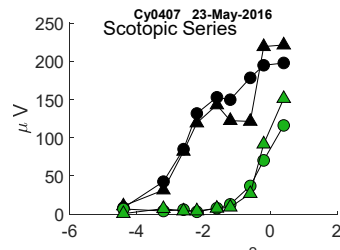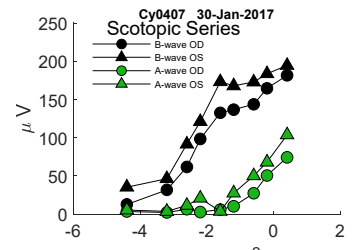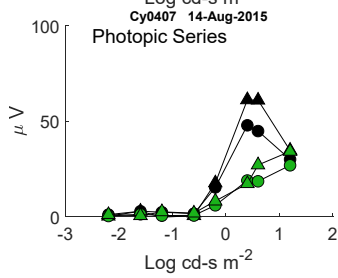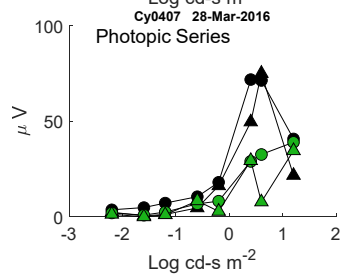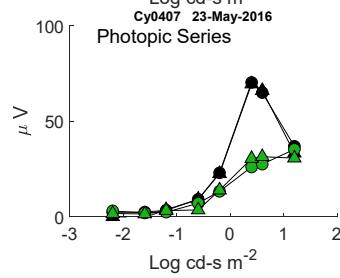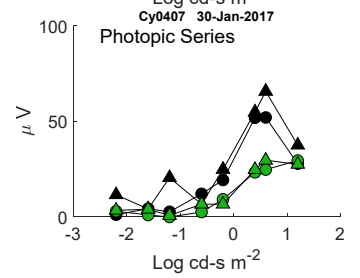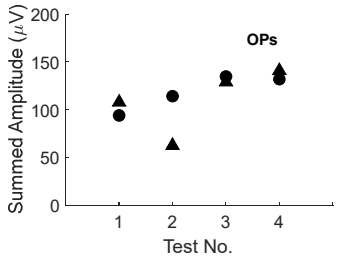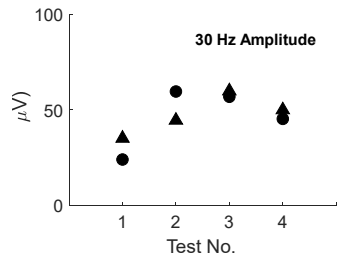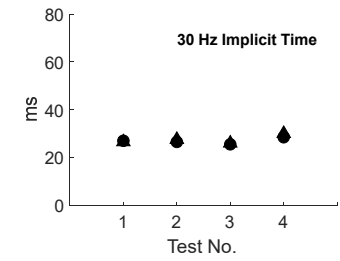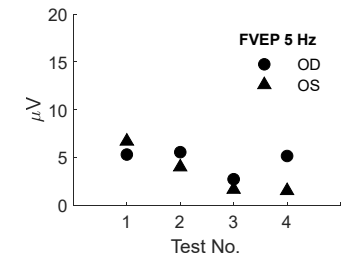

Supplement: S1 Fig — Representative ffERG data acquired under dark adaptation (scotopic series) and light adaptation (photopic series), Oscillatory potentials (OPs) data, 30.3-Hz flicker ERG amplitude and timing data and 5.0-Hz flash visual evoked potentials (FVEP) amplitude data from NHP Cy0407 are presented. Rod-mediated and cone-mediated A- and B-waves exhibited normal increases with flash strength, consistent with preserved outer (photoreceptors) and inner (bipolar cells; Müller cells) function. Generally, lower amplitudes were elicited following the first test, but scotopic and photopic ERG amplitudes remained relatively constant for the remainder of the study for all 3 animals. No instances of ‘flat’ or extinguished ERGs were noted. OPs amplitude, 30-Hz flicker ERG amplitude and timing and FVEP amplitude were unaltered across the course of the studies. No clear or consistent evidence of physiologically significant changes in retinal or retino-cortical function were evident in any of the 3 NHPs tested. (PDF) [file pone.0313579.s001.pdf]

Sens 100

Sens 90

Sens 80

Sens 70

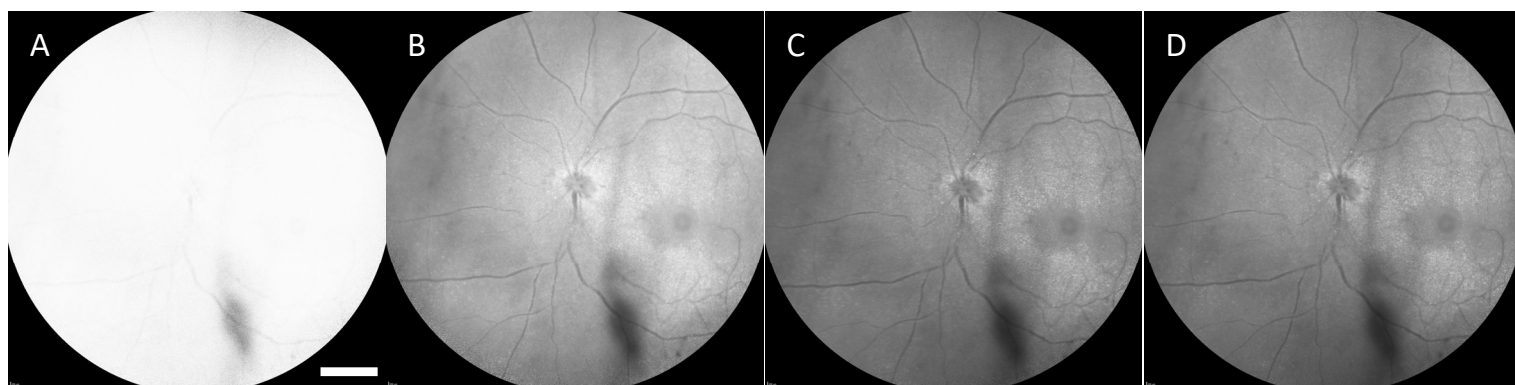

Supplement: S2 Fig — Numbers 100, 90, 80 and 70 were total sensitivity settings for each image below the number, respectively. The haze from 12 nmol TcapQ488 injection was confirmed by all total sensitivity settings from the vitreous obscuring fluorescence signals from GCL. Fluorescence angiography mode (FAM), Oculus Sinister (OS), Ganglion cell layer (GCL), Scale bar (2 mm). (PDF) [file pone.0313579.s002.pdf]

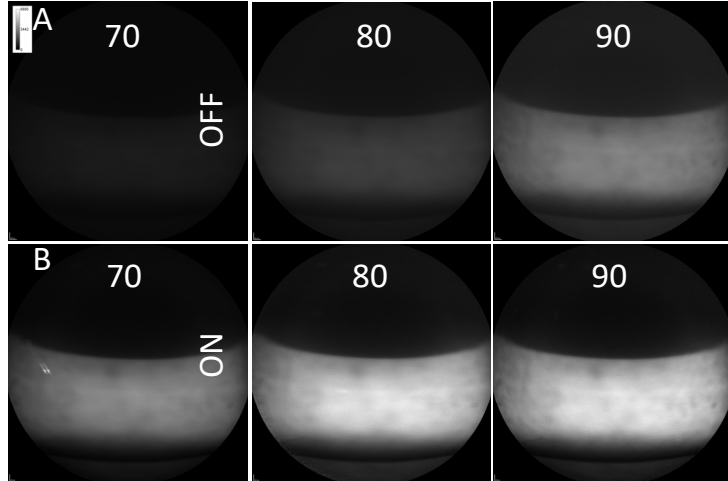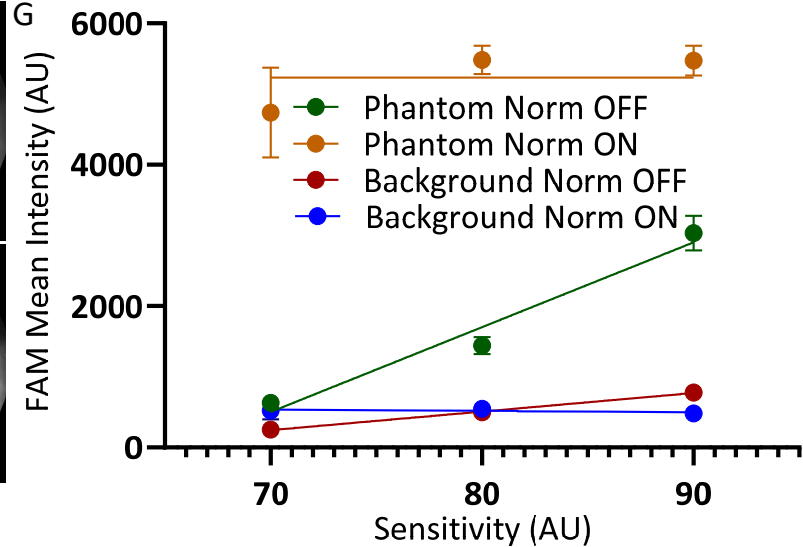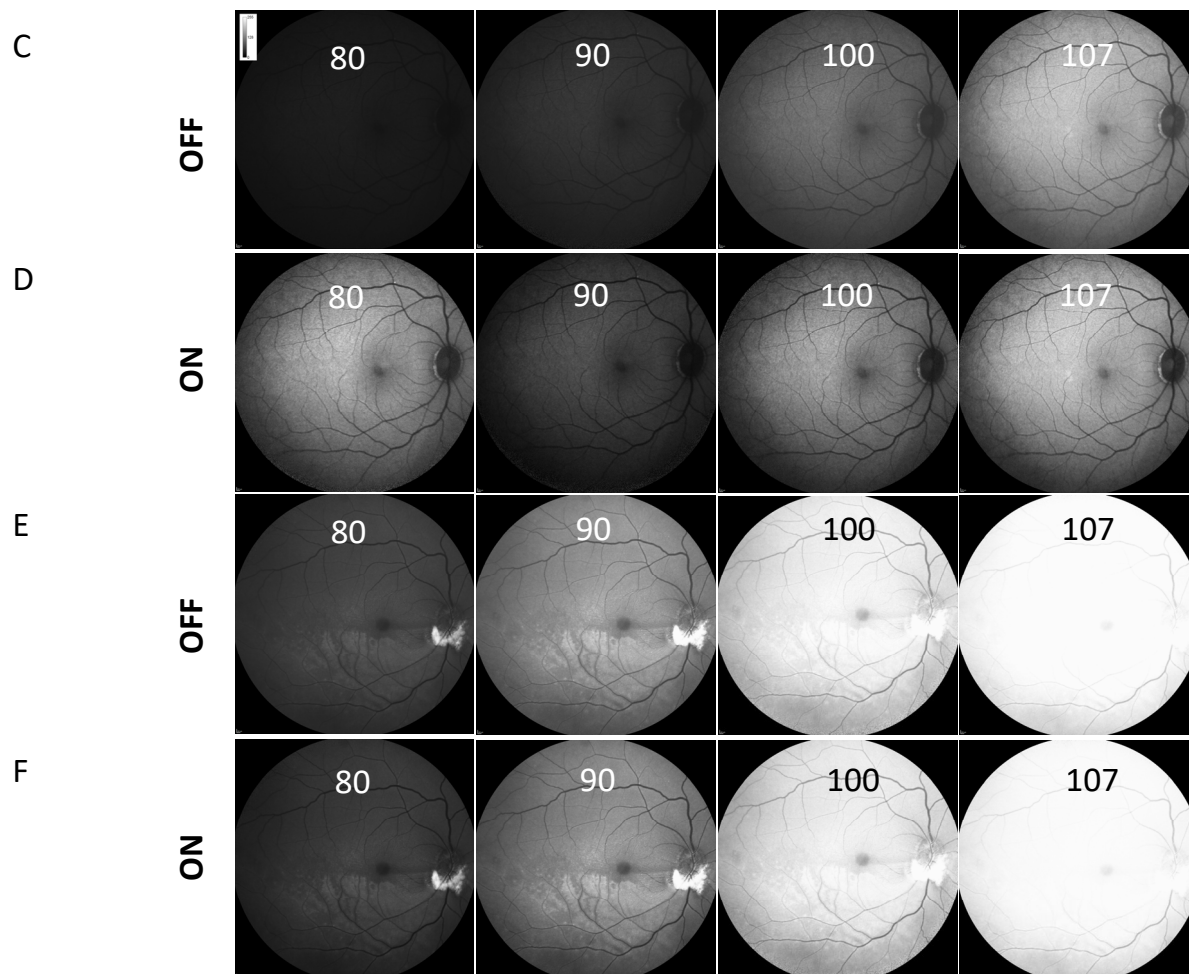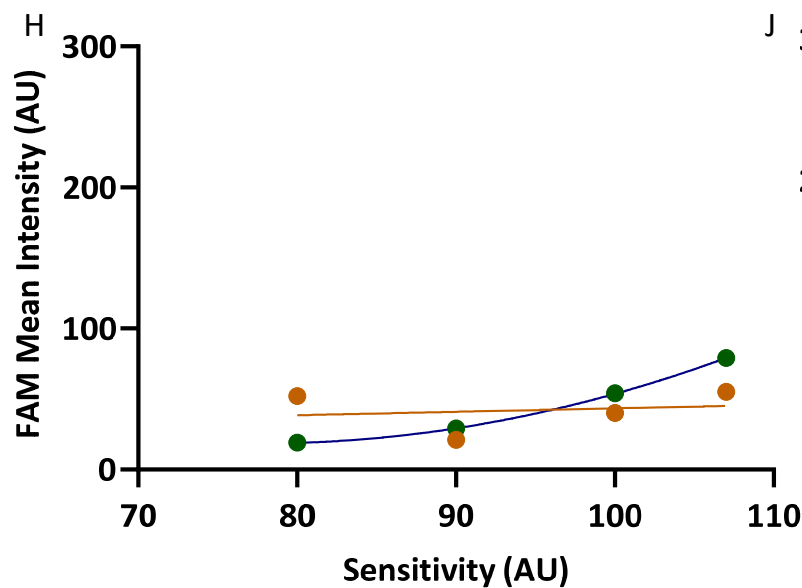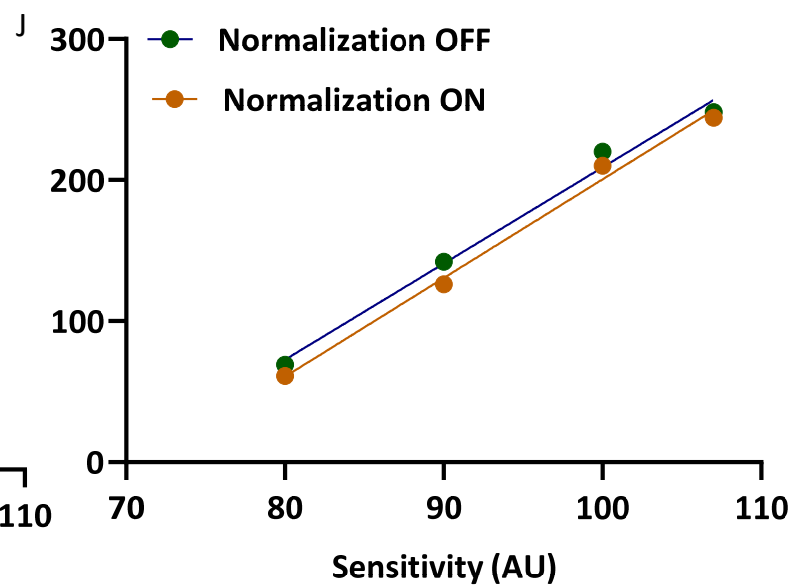

Supplement: S3 Fig — (PDF) [file pone.0313579.s003.pdf]

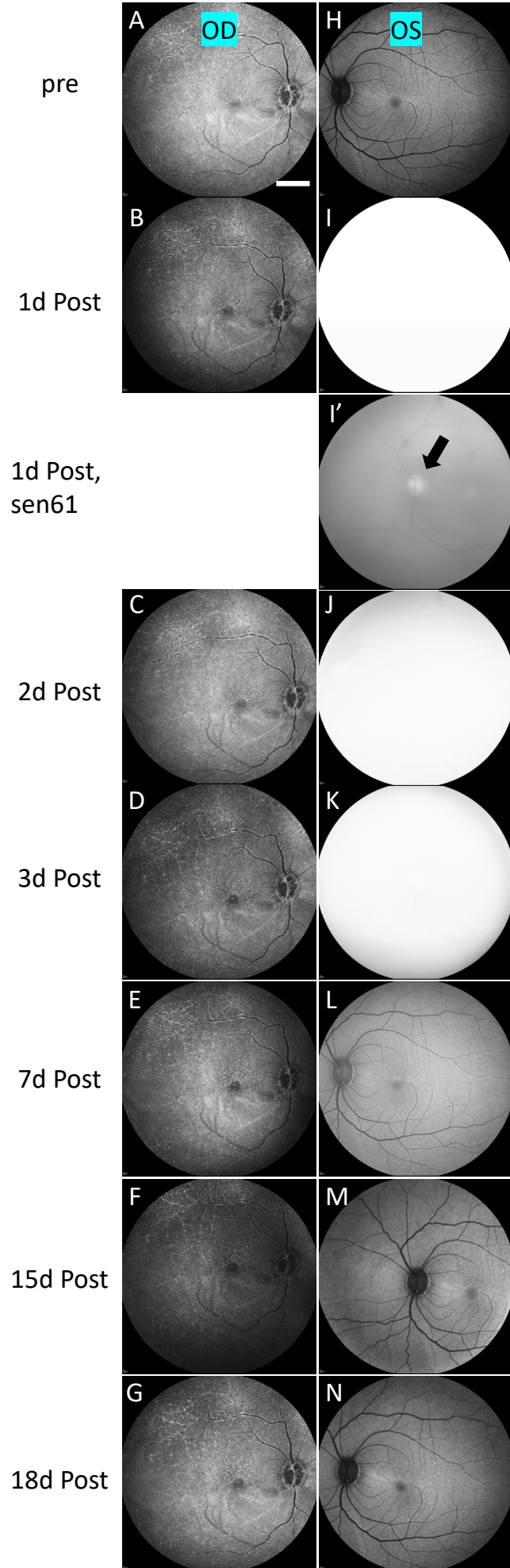

Supplement: S4 Fig — Longitudinal Spectralis FAM images of Cy0410 OD (A-G) and OS (H-N) were paired based on time of non-cleavable, non-quenched 6 nmol TAT peptide probe dTcap488 intravitreal injection. Punctate fluorescent signals detected at OD were from previous HEA procedure and consecutive TcapQ488 intravitreal injections (A-G) to OD. I’) one day post the probe injection when total sensitivity setting was at 61 (all other images were set at 100). Arrow pointed hyperfluorescent signal at ONH, suggesting dTcap488 was actively transported towards the brain via RGC axons. The dTcap488 haze stayed in the vitreous for approximately 15 days before being cleared out. At Post 15 days (M) and 18 days (N) post dTcap488 injection, there was few detectable fluorescent signals at GCL, and the ONH was fluorescently hypo labeled. Scale bar (2 mm). (PDF) [file pone.0313579.s004.pdf]
