# Supplementary material for: In vivo scanning laser fundus and high-resolution OCT imaging of retinal ganglion cell injury in a non-human primate model with an activatable fluorescent-labeled TAT peptide probe
Source: PLoS One. 2024 Dec 6;19(12):e0313579. doi: 10.1371/journal.pone.0313579 (PMC11623487; doi:10.1371/journal.pone.0313579)
Supplement: S1 File — (DOCX) [file pone.0313579.s005.docx]

**WIMR Housing conditions**

The WNPRC houses macaques living at their Wisconsin Institutes for Medical Research (WIMR) vivarium in traditional stainless steel mobile enclosures capable of housing 1-4 animals (e.g., “two-packs, “four-packs”). Per AWA regulations, each enclosure of the two- and four-bank units have at least 4.3, 6.0, or 8.0 sq. ft. of floor space, and measure 30, 32, or 36 inches high, and contain a tubular PVC or stainless-steel perch to allow an animal to utilize the vertical dimensions of the enclosure.  Each enclosure of the two- and four-bank units is equipped with a horizontal or vertical sliding door so that multiple living configurations can be created out of a single row or bank of enclosures., These sliding doors allow animals to be paired or to be placed in even larger social groups to enhance psychological health and to inspire species-typical behavior (e.g., grooming, play, allo-parenting, etc.).

**Feeding regimens**

The nutritional plan utilized by the WNPRC is based on recommendations published by the National Research Council and the input of WNPRC investigators with expertise in nonhuman primate nutrition and metabolism.  A majority of the macaque colony is fed with 2050 Teklad Global 20% Protein Primate Diet (Teklad Diets, Madison, WI). The commercial NHP diet fed to the macaque colony is also supplemented with a variety of fruits, vegetables, and nuts. All diets are fed as outlined in WNPRC SOP 1.05 (Animal Feeding and Watering) and support animals in all life stages (i.e., neonate to geriatric).  Feeding strategies are additionally tailored for the physical condition of each member of the colony (e.g., pregnancy, lactation, cachexia due to disease, colitis/malabsorption, obesity, etc.).  Each animal caretaker is trained to closely monitor the intake of the animals they are responsible for and to communicate this information to the veterinary staff on a frequent basis to ensure that necessary changes in the amount or type of chow offered is made in a timely fashion.

**Environmental Enrichment**

The WNPRC Behavioral Services Unit promotes animal welfare and facilitates research by providing state-of-the-art behavioral management of the macaques housed at WNPRC. The unit is supervised by a PhD level behaviorist and consists of an enrichment coordinator and three behavioral technicians who work on a daily basis to implement the WNPRC Environmental Enhancement Plan (EEP). The work of the unit is driven by the overriding appreciation that the best animal models are those that are psychologically well adjusted. One of the principles of the EEP is to provide an enhanced captive environment that prevents or reduces the development of abnormal behavior and promotes active engagement in species-typical behavior.  As noted in Section I.C., the main component of the EEP is a socialization program, especially focused on the WNPRC’s macaque colonies. In addition to socialization, the EEP also includes additional strategies to further promote species-typical behavior and to decrease expression of abnormal/stereotypic behavior including the provision of a wide variety of foraging devices, manipulanda (e.g., Kong toys, dental balls, Nyla rings, plastic dumbbells, etc.), and structural enhancements (e.g., perches, logs, hammocks, ropes, ladders, privacy panels, etc.) to each nonhuman primate enclosure at the WNPRC.  To ensure the efficacy of the EEP, animals are monitored for abnormal behavior on a frequent basis.  Animals exhibiting abnormal behavior are treated accordingly and the EEP is continuously reviewed and adjusted to maintain/increase positive outcomes.

**How often animal care staff monitored the health and well-being of the animals and the criteria used to make such assessments**

To ensure the health of the WNPRC nonhuman primate colony, each animal is evaluated twice daily by an animal research technician or veterinary technician for the evidence of disease or injury (e.g., inappetence, dehydration, diarrhea, depression, inactivity, trauma, etc.).  Using an iPad, the technician generates a daily report of animals that need veterinary attention, and the veterinary staff evaluates each animal and treats them accordingly.  A current problem and treatment list is updated daily by the veterinary technicians and veterinarians to ensure that all clinical problems are treated appropriately.  All clinical problems, treatments, and case outcomes are entered into the WNPRC electronic health records database so that complete histories can be generated, and ongoing clinical problems can be tracked.

**Standard euthanasia**

If required, animals were euthanized by administering an intramuscular dose of with ketamine (15 mg/kg) followed by an intravenous overdose (50 mg/kg or to effect) of sodium pentobarbital.  This method is in compliance with the American Veterinary Medical Association’s 2020 Guidelines for the Euthanasia of Animals.

**Perfused animals**

If animals were perfused, prior to perfusion, animals were premedicated with an intramuscular (IM) dose of with ketamine (15 mg/kg)) and hydromorphone (0.05 - 0.2 mg/kg, IM) followed by a deep surgical plane of anesthesia induced with intravenous sodium pentobarbital (at least 35 mg/kg).  Deep pain, palpebral, and corneal reflexes were checked to verify an adequate plane of anesthesia and  then the sternum of the animal was reflected cranially utilizing sharp dissection and rib cutters. The descending thoracic aorta was then isolated and occluded by clamping with 1-2 pairs of hemostats. The pericardium was incised to visualize the heart, the left ventricle was visualized and cannulated, and the right auricle was transected. The animal was perfused via the left ventricle, first with physiological saline to remove red blood cells from the tissues, followed by perfusion with cold phosphate-buffered saline containing an appropriate fixative.
